# A Lightweight Deep Learning Model for Fast Electrocardiographic Beats Classification With a Wearable Cardiac Monitor: Development and Validation Study

**DOI:** 10.2196/17037

**Published:** 2020-03-12

**Authors:** Eunjoo Jeon, Kyusam Oh, Soonhwan Kwon, HyeongGwan Son, Yongkeun Yun, Eun-Soo Jung, Min Soo Kim

**Affiliations:** 1 Technology Research Samsung SDS Seoul Republic of Korea

**Keywords:** path-type ECG sensor system, ECG classification, deep learning, recurrent neural network, fused recurrent neural network

## Abstract

**Background:**

Electrocardiographic (ECG) monitors have been widely used for diagnosing cardiac arrhythmias for decades. However, accurate analysis of ECG signals is difficult and time-consuming work because large amounts of beats need to be inspected. In order to enhance ECG beat classification, machine learning and deep learning methods have been studied. However, existing studies have limitations in model rigidity, model complexity, and inference speed.

**Objective:**

To classify ECG beats effectively and efficiently, we propose a baseline model with recurrent neural networks (RNNs). Furthermore, we also propose a lightweight model with fused RNN for speeding up the prediction time on central processing units (CPUs).

**Methods:**

We used 48 ECGs from the MIT-BIH (Massachusetts Institute of Technology-Beth Israel Hospital) Arrhythmia Database, and 76 ECGs were collected with S-Patch devices developed by Samsung SDS. We developed both baseline and lightweight models on the MXNet framework. We trained both models on graphics processing units and measured both models’ inference times on CPUs.

**Results:**

Our models achieved overall beat classification accuracies of 99.72% for the baseline model with RNN and 99.80% for the lightweight model with fused RNN. Moreover, our lightweight model reduced the inference time on CPUs without any loss of accuracy. The inference time for the lightweight model for 24-hour ECGs was 3 minutes, which is 5 times faster than the baseline model.

**Conclusions:**

Both our baseline and lightweight models achieved cardiologist-level accuracies. Furthermore, our lightweight model is competitive on CPU-based wearable hardware.

## Introduction

### Background

Arrhythmia refers to any change causing the heart to beat too fast or slow, or erratically [1], and can lead to sudden death or critical adverse outcomes such as embolic stroke [2]. Therefore, early detection and treatment of arrhythmia are very important.

One of the most widely used diagnostic methods for detecting arrhythmia is electrocardiographic (ECG) monitoring. ECG monitoring is a simple and noninvasive method for recording electrical activities of the heart by using electrodes placed on human skin. However, at least 24 hours of ECG signals should be monitored to confirm arrhythmia since it occurs irregularly [3,4]. Recently, single lead patches that are wireless, compact, and lightweight have been proposed for long-term wear [5-7]. Despite improvements to measuring ECGs and patient comfort, it is still difficult to diagnose arrhythmias because identification of abnormal ECG patterns from large amounts of recorded ECGs is not trivial. For example, an ECG record, measured for 24 hours in patients with a heart rate of 80 bpm, consists of 110,000 beats. It takes at least 2 hours for an expert to analyze this 24-hour ECG signal.

Large-scale machine learning methods have been investigated to reduce the human efforts for ECG beat classification [8-10]. However, most machine learning approaches with static and handcrafted features have performed at lower accuracy rates over new types of ECGs because those features are insufficient for representing the great diversity of ECG patterns from various patients. Therefore, several self-learning approaches based on a deep neural network have been proposed recently [11-13].

Among the deep learning approaches, convolutional neural networks (CNNs) and recurrent neural networks (RNNs) are most commonly used for ECG classification. CNNs typically consist of convolution, pooling, and fully connected layers [14]. CNNs extract implicit features of ECGs through each level of convolution layer and use the abstraction from these features to solve problems such as classification and regression [15]. Rajpurkar et al [11] used a CNN to classify 12 ECG rhythms, which are longer units consisting of 2 or more beats. Their model consisted of 33 convolutional layers with shortcut connections followed by a fully connected layer and a softmax layer. The model achieved an F1 score of 0.81 compared with the responses of board-certified cardiologists. Acharya et al [12] proposed an ECG beat classification model using a CNN together with noise removal, wavelet transformation, and segmentation method. Their model consisted of three convolutional layers, three max-pooling layers, three fully connected layers, and finally, a softmax layer with five output neurons. The model resulted in an average accuracy of 94.03% compared with the MIT-BIH (Massachusetts Institute of Technology-Beth Israel Hospital) gold standard.

However, a limitation of CNNs is that the length of inputs must be fixed since the filters of the networks have static sizes. When it comes to ECG classification, the length of ECGs can be varied according to an individual’s heart rate. Therefore, adjusting data such as linear interpolation is required to achieve same-size inputs [11-13].

In contrast, RNNs are able to handle this sequential problem because the networks recursively learn data as time progresses [16]. Tan et al [17] proposed the implementation of a long short-term memory network (LSTM), which is the most widely used method among RNN approach, with a CNN to diagnose the presence of coronary artery disease from the ECG signals. Although they focused on specific diseases, they achieved an F1 score of 0.96. Oh et al [18] diagnosed five types of rhythms: normal sinus rhythm, left bundle branch block, right bundle branch block, atrial premature beats, and premature ventricular contraction. Their model consisted of three 1D convolution layers, one LSTM layer, and three fully connected layers. They achieved a 98.10% accuracy using 10-fold cross-validation. Yildirim [19] also classified five types of rhythms but chose bidirectional LSTM (bi-LSTM) instead of unidirectional LSTM (uni-LSTM). The proposed model was composed of four wavelet transform layers, two bi-LSTMs, and two fully connected layers. This model showed a recognition accuracy of 99.39%.

However, existing studies using RNNs have limitations in application [17-20]. First, subject-specific evaluation to explore differences between patients is generally not conducted. Therefore, it is difficult to trust predictions of RNNs on new patients’ ECG signals that were not included in the training data. Second, RNNs have disadvantages related to financial cost and inference time. Most of the papers did not consider the cost of using a graphics processing unit (GPU) instead of a central processing unit (CPU) and did not present the time to inference with their deep learning models. This weakness in computational efficiency is a critical drawback of RNN applications. To accelerate and maximize the computational efficiency of RNN layers, MXNet proposed fused RNN operator by applying several optimization methods: (1) various general matrix multiplication (GEMM) modes such as combining small GEMMs, Batch GEMM, and Pack GEMM; (2) vectorization of elementwise operations using Basic Linear Algebra Subprogram (BLAS) libraries and Intel Math Kernel Library (MKL); and (3) saving and reusing intermediate results during forward computation [21,22].

### Objectives

We used ECG signals measured with Samsung S-Patch 2, a small (120×29×4.4 mm in size) and light (8 g in weight) patch-type ECG monitor [7]. To diagnose arrhythmias using S-Patch devices effectively and efficiently, we propose a baseline model with RNN that can learn sequential patterns. Furthermore, we also propose a lightweight model with fused RNN for conducting the classification process on CPUs with a shorter prediction time.

## Methods

### Data Collection

We analyzed an open-source ECG database (PhysioBank MIT-BIH Arrhythmia Database [23]) together with our own deidentified dataset collected with the S-Patch device. Overall, the MIT-BIH Arrhythmia Database contains 48 subjects’ ECGs, each measured for 24 hours at 360 Hz.

**Figure 1:**
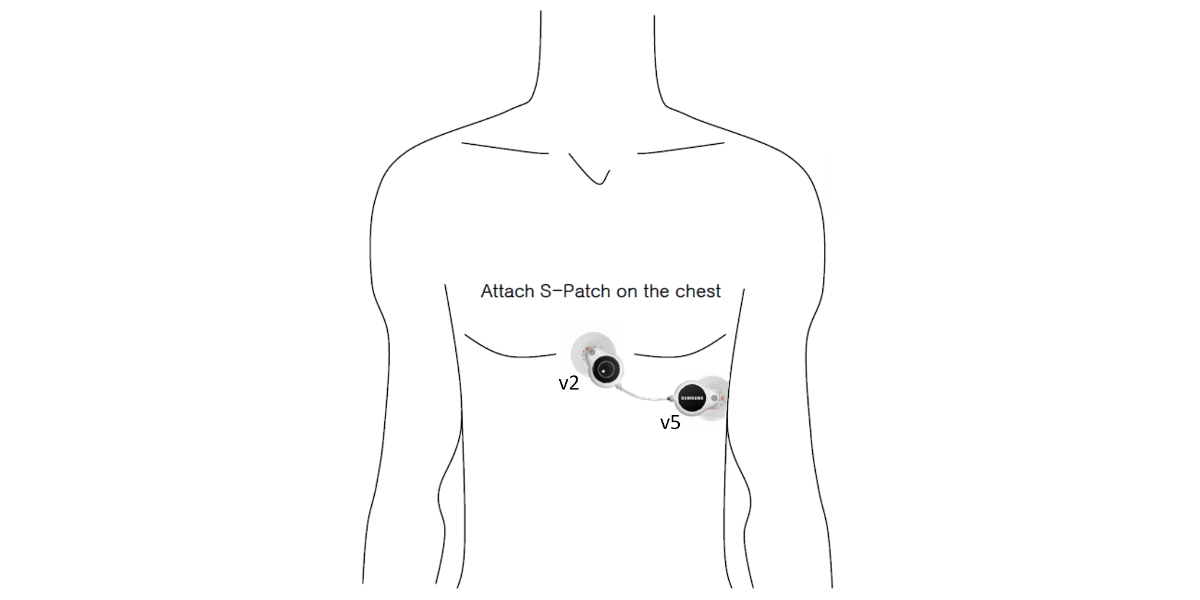
Usage of S-Patch for Samsung SDS Cardio.

The S-Patch database was obtained according to the following procedures. First, we collected the ECGs using S-Patch at the Samsung Medical Center in Seoul and at Counties Manukau Health in New Zealand from February 2017 to April 2018. A skilled nurse at each hospital attached an ECG monitor and checked whether the ECGs were normally collected over the first 5 minutes. The patches of S-patch cardiac monitor were attached on v2 and v5 positions of the 12-lead placement (Figure 1). Second, three experts, who had more than 5 years of experience working in a territorial hospital, reviewed each record and excluded ones that contained noise levels of more than 80% to enhance the data quality. Subsequently, we anonymized data by removing personal and location information. Third, the three experts annotated each beat using the Web portal. If consensus could not be reached on the classification of a beat, the experts rediscussed the issue to make a final decision. Consequently, we collected 1828-hours of ECG data from 76 subjects. The average length of the ECGs from S-Patch was 17 hours (from 28 minutes to 45 hours). Each ECG collected with S-Patch was sampled at 256 Hz.

In this study, we used five beat categories defined in the AAMI/IEC (Association for the Advancement of Medical Instrumentation / International Electrotechnical Commission) standard [24] (ie, nonectopic [N], supraventricular ectopic [S], ventricular ectopic [V], fusion [F], and paced or unknown [Q]). Overall, 5,575,512 ECG beats were used in this study, as shown in Table 1.

**Table 1:** The five subtype classes and the number of samples.

AAMI^a^/IEC^b^ categories	Number of beats	
	MIT-BIH^c^ dataset, n (%)	S-Patch dataset, n (%)
Nonectopic	90,386 (82.19)	5,303,245 (97.03)
Supraventricular ectopic	3026 (2.75)	27,288 (0.5)
Ventricular ectopic	7708 (7.01)	135,013 (2.47)
Fusion	803 (0.74)	0 (0)
Paced or unknown	8043 (7.31)	0 (0)
Total	109,966 (100.00)	5,465,546 (100.00)

^a^AAMI: Association for the Advancement of Medical Instrumentation.

^b^IEC: International Electrotechnical Commission.

^c^MIT-BIH: Massachusetts Institute of Technology-Beth Israel Hospital.

### Data Preprocessing

**Figure 2:**
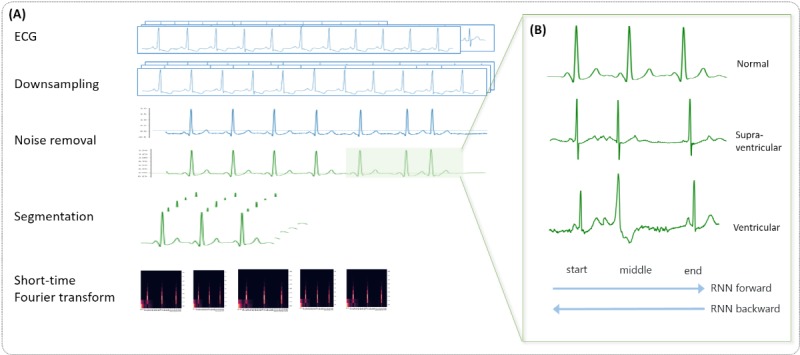
Data preprocessing of electrocardiograms: (A) full steps from downsampling to short-time Fourier transform; (B) an example of a 3-beat electrocardiographic segment for each class. ECG: electrocardiogram; RNN: recurrent neural network.

We performed ECG preprocessing as follows (Figure 2A): downsampling, noise removal, segmentation, and short-time Fourier transform (STFT). The examples of preprocessed signals for the beat classes are also depicted (Figure 2B): A normal beat has a regular beat interval with a small wave (P-wave) before a larger and sharper wave (QRS wave); a supraventricular beat has an irregular beat interval; a ventricular beat has a wide QRS wave with a vague or no P-wave; a fusion beat is a combined pattern of normal and ventricular beat; and a paced or unknown beat has none of the abovementioned features and can be observed in diverse patterns.

**Figure 3:**
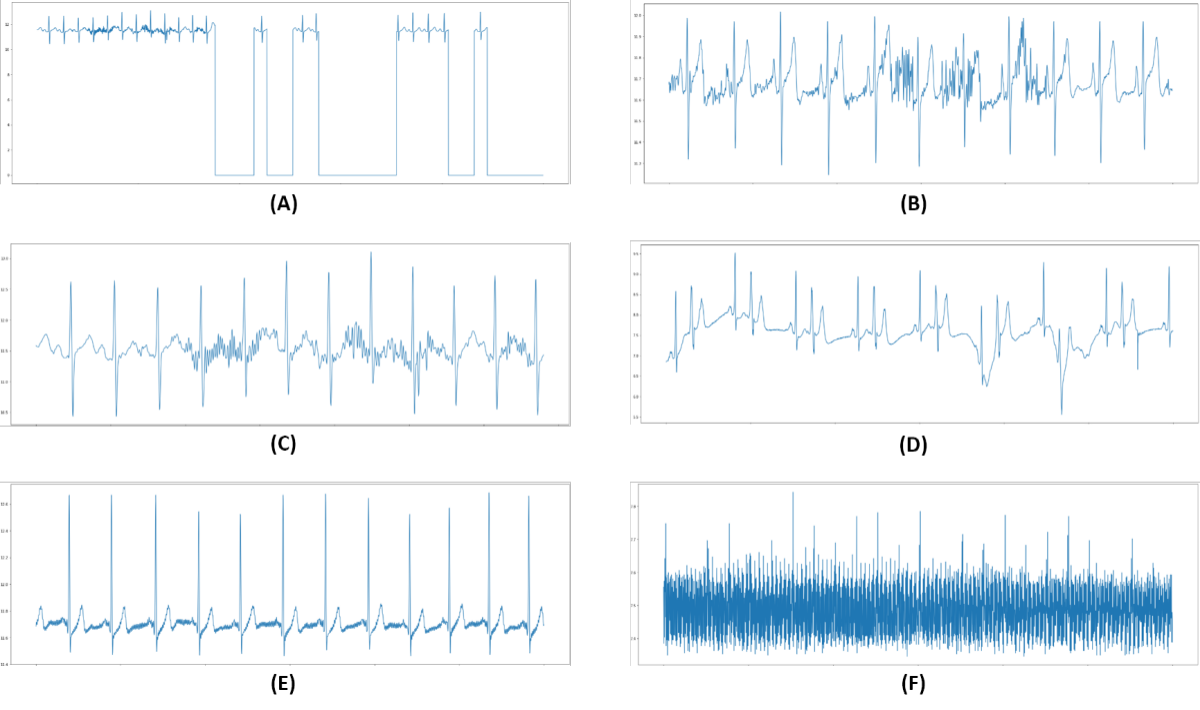
Examples of electrocardiographic signal noise: (A) loose contact, (B) motion artifact, (C) muscular activation interference, (D) baseline wandering, (E) alternating current (AC) interference (low signal-to-noise ratio), (F) AC interference (high signal-to-noise ratio).

First, we downsampled the ECG signals to handle the different sampling rates between data from difference databases. For consistency, the MIT-BIH records were downsampled to 256 Hz, which is the same as the sampling rate of the S-Patch dataset. Second, we tried to reduce artifacts in the data. The ECGs collected with S-Patch are real-world data. Therefore, they contained all kinds of noise such as loose contacts, motion artifact, muscular activation interference, baseline wandering, and AC (alternating current) interference (Figure 3). We excluded noise caused by loose contact that falls below 0 mV. Thereafter, we applied a bandpass filter with a high-frequency cutoff at 40 Hz and a low-frequency cutoff at 0.5 Hz to handle other types of noise. Third, in order to deal with 24-hour ECGs effectively, we segmented the ECGs into beat units. Since the duration of a beat is different according to the heart rate of each patient, we extracted 3-beat ECG signals with R-peaks identified using the algorithm developed by Kathirvel et al [25] instead of an arbitrary time duration. Specifically, we selected a window with a length of 3 beats because information on the middle beat, which is the target to be classified, is affected by the preceding and following beats. Finally, we applied STFT to all ECG segments.

### Baseline Model With Recurrent Neural Network

**Figure 4:**
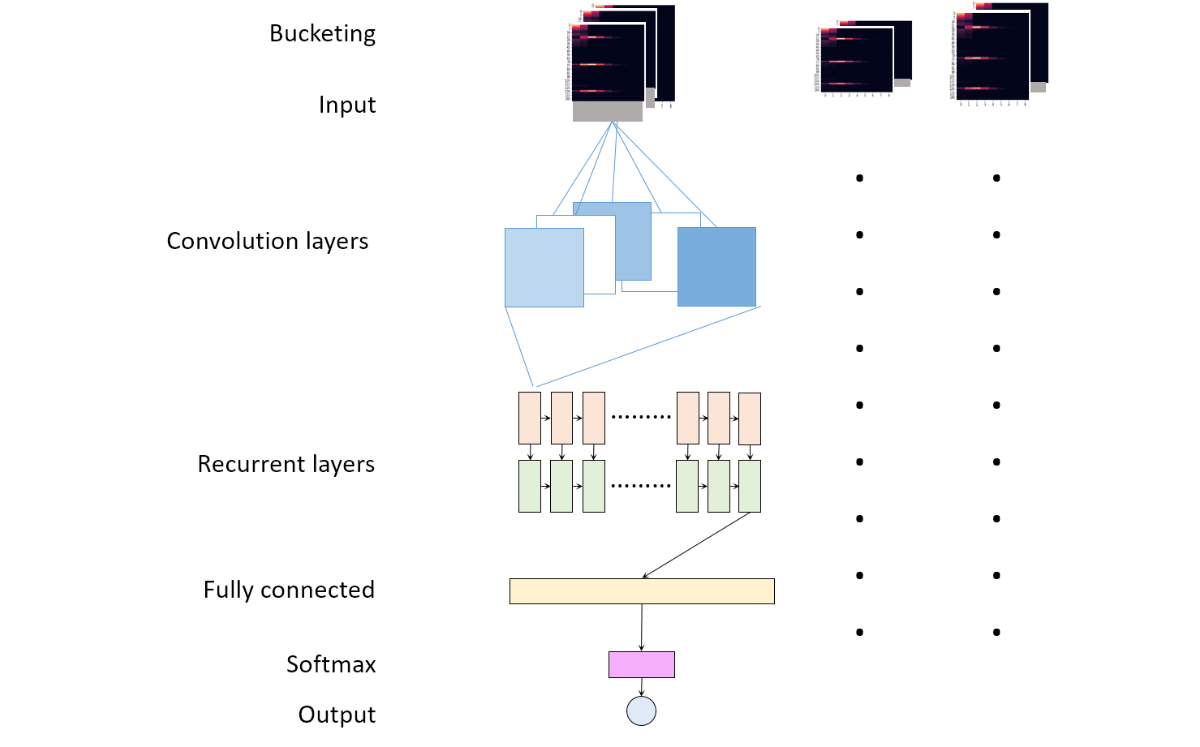
Model architecture (general).

We segmented ECG signals into 3-beat units during the preprocessing; thus, their lengths varied according to the subjects’ heart rates. As baselines for ECG pattern classification, we implemented Vanilla RNNs (Figure 4), which can handle input sequences with various lengths. However, the variable length of input data can reduce the learning efficiency of deep learning models. Therefore, we used a bucketing method to handle the variable length of inputs. Bucketing is suggested to improve the parallelization capabilities of the recurrent training process. We set up several buckets and assigned each instance to the bucket with the closest size. Within a bucket, each instance was padded with zeroes up to the length of the bucket. Although the buckets had different internal models, their parameters were shared in time.

After bucketing, the training process for instances of each bucket was as follows: first, the input data was convolved with 11 filters (3×11 in size) with a stride of 1 in the first convolution layer, which was followed by a convolution layer with 11 filters (3×3 in size) with a stride of 1. Second, the outputs of the convolution layer proceeded through consecutive two Vanilla RNN layers with hidden states of 1760 for each. Finally, the outputs of the RNN layers passed to a fully connected layer, and a softmax function with 5 output nodes was used in the final layer. Batch normalization was used in each layer of the architecture.

### Lightweight Model With Fused Recurrent Neural Network

Baseline model inference was performed on CPUs (Intel Xeon Platinum 8000 v4). However, it was about 6 times slower than the GPU-based inference (Tesla K80). In order to improve the inference speeds on CPUs, we propose a lightweight model by reducing the input size and adopting fused RNN (Table 2).

To reduce the input size, we selected a minimum sampling rate by halving the sampling rate from 256, provided there was no degradation in accuracy. Finally, we downsampled the MIT-BIH (360 Hz) and S-Patch data (256 Hz) to 64 Hz. As the input size decreased, the filter size changed from 3×11 to 3×5 and the number of convolution layers changed from two to one, compared to the baseline model. Additionally, we changed the RNN layers to fused RNN instead of Vanilla RNN to maximize the computation efficiency of the RNN layers in CPUs. Additionally, we used Intel Math Kernel Library 2018 update 3 for matrix-multiplication operation.

**Table 2:** Comparison of the baseline and lightweight models.

Characteristic	Baseline model	Lightweight model
Sampling rate (Hz)	256	64
Convolution layer	2	1
Convolution filter size	3×11×11, stride 1; 3×3×11, stride 1	3×5×11, stride 1
Recurrent layer	Vanilla RNN^a^	Fused RNN

^a^RNN: recurrent neural network.

### Experimental Setup

We divided a total of 124 subjects into two groups—112 and 12 subjects for the train (including validation) and test sets, respectively. The train set consisted of 43 subjects from MIT-BIH and 69 from S-Patch, and the test set consisted of 5 subjects from MIT-BIH and 7 from S-Patch. Specifically, the 12 subjects in the test set were carefully selected by cardiologists to evaluate various types of beats.

Normal beats comprised more than 90% of the total data; therefore, we randomly sampled beats in the normal class equal to the total number of beats in other classes every epoch to avoid this data imbalance problem. Moreover, most of the samples for abnormal classes were from MIT-BIH; thus, data imbalance between MIT-BIH and S-Patch were also handled by balancing the number of samples for normal and other classes.

We used MXNet to create the baseline model with RNN and the lightweight model with fused RNN [26]. We trained both models on GPUs (OS: Linux, CPU: Intel Xeon E5-2686 v4 processor, memory: 488 GB, GPU: four NVIDIA K80 GPU) with Xavier initialization and Adam optimizer. The baseline model’s learning rate was 5E-06 with a batch size of 1000 over 400 epochs, and the lightweight model’s learning rate was 1E-05 with a batch size of 900 over 300 epochs. After training, we selected the best model with the highest validation accuracies for the three classes (N, S, and V Classes).

### Evaluation Metric

Classification performance was measured by four standard metrics (ie, accuracies, sensitivities, specificities, and positive predictive values) that have been used in the literature [9,12,13]. These were calculated using the four values from the confusion matrix, true positive (TP), true negative (TN), false positive (FP), and false negative (FN). The accuracy is the ratio of the number of correctly classified patterns to the total numbers of patterns classified, 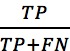. The sensitivity is the rate of correctly classified events among all events, 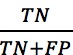. The specificity is the rate of correctly classified nonevents among all nonevents, 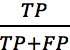. The positive predictive value is the rate of correctly classified events in all detected events, 
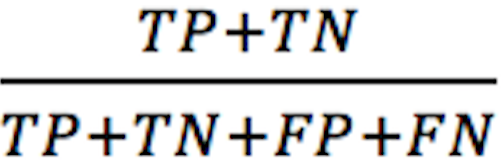
. In addition to the metrics, we calculated an overall accuracy using the equation proposed by Landis and Koch [27]. To measure the inference speed, 24-hour ECGs with 64,976 beats were inferenced on both the GPUs and CPUs (OS: Linux, CPU: eight 3.0 GHz Intel Xeon Platinum processors, memory: 16 GB).

## Results

### Accuracy of Baseline and Lightweight Models

**Figure 5:**
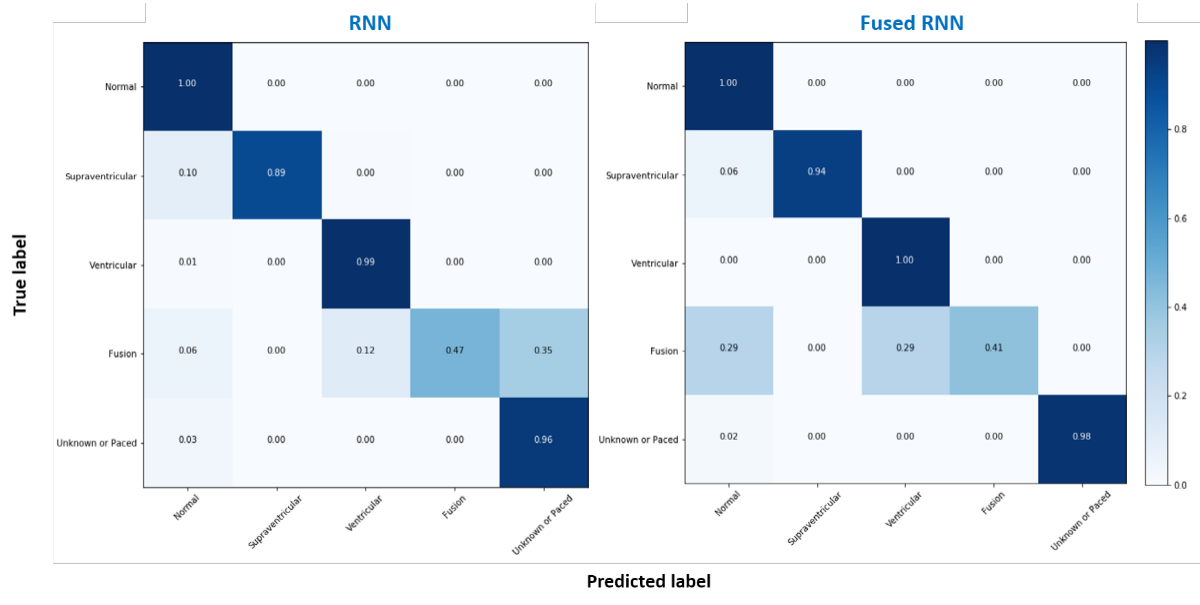
Normalized confusion matrix of the recurrent neural network (RNN) and fused RNN models.

Figure 5 shows the overall performances in the classification of ECGs for the baseline (RNN) and lightweight (fused RNN) models. There was no significant difference between the baseline and lightweight models because the overall accuracies were close to each other: 99.72% for baseline and 99.80% for the lightweight model.

Tables 3 and 4 demonstrate the accuracies of the baseline and lightweight models for Class S (99.82% and 99.90%, respectively) and for Class V (99.91% and 99.89%, respectively). These results were far superior to those of previous works, wherein the results ranged from 92.4% to 97.6% for Class S and from 96.7% to 99.0% for Class V [13,28,29]. Note that we excluded the results for Class F because the number of beats in Class F was only 17.

Furthermore, both baseline and lightweight models performed higher than 97% in subject-specific accuracies, similar to the overall accuracy (Table 5). These results ensured the internal reliability of the models. The accuracy fluctuation range was 2.42% for baseline (from 97.56% to 99.88%) and 0.84% for lightweight model (from 99.12% to 99.96%). Our lightweight model with fused RNN improved overall accuracy and internal reliability even though it is relatively lighter.

**Table 3:** A confusion matrix of the baseline model for the test set.

Class (ground truth)	Classification results								
	Predicted class					ACC^f^ (%)	PPV^g^ (%)	SEN^h^ (%)	SPEC^i^ (%)
	n^a^	s^b^	v^c^	f^d^	q^e^				
N	481,491	362	215	2	46	99.75	99.87	99.86	98.31
S	438	3935	20	0	5	99.82	89.47	89.17	99.91
V	172	116	33,980	5	11	99.90	99.11	99.29	99.95
F	1	0	2	8	6	N/A^j^	N/A	N/A	N/A
Q	71	0	5	0	1984	99.97	96.31	96.69	99.99

^a^n/N: nonectopic.

^b^s/S: supraventricular ectopic.

^c^v/V: ventricular ectopic.

^d^f/F: fusion beat.

^e^q/Q: paced or unknown beat.

^f^ACC: accuracy.

^g^PPV: positive predictive value.

^h^SEN: sensitivity.

^i^SPEC: specificity.

^j^Not applicable.

**Table 4:** A confusion matrix of the lightweight model for the test set.

Class (ground truth)	Classification results											
	Predicted class						ACC^f^ (%)	PPV^g^ (%)		SEN^h^ (%)		SPEC^i^ (%)
	n^a^	s^b^	v^c^	f^d^	q^e^							
N	481,587	130	403	1	7	99.83		99.89	99.92		99.11	
S	264	4123	6	0	0	99.91		93.85	94.80		99.96	
V	51	96	34,157	7	0	99.89		99.55	98.80		99.92	
F	5	0	5	7	0	N/A^j^		N/A	N/A		N/A	
Q	43	0	0	0	2017	99.99		97.91	99.65		100.00	

^a^n/N: nonectopic.

^b^s/S: supraventricular ectopic.

^c^v/V: ventricular ectopic.

^d^f/F: fusion beat.

^e^q/Q: paced or unknown beat.

^f^ACC: accuracy.

^g^PPV: positive predictive value.

^h^SEN: sensitivity.

^i^SPEC: specificity.

^j^Not applicable.

**Table 5:** Overall accuracies of the baseline and lightweight models according to subjects in the test set. (Subject numbers 100 to 223 are from MIT-BIH, and the rest are from S-patch.)

Subject #	Rhythm	Beats, n	RNN^a^				Fused RNN			
			ACC^b^ (%)	PPV^c^ (%)	SEN^d^ (%)	SPEC^e^ (%)	ACC (%)	PPV (%)	SEN (%)	SPEC (%)
100	Normal	2273	99.74	99.34	99.34	99.84	99.91	99.78	99.78	99.95
104	Paced	2225	98.04	95.10	95.10	98.78	99.12	97.80	97.80	99.45
108	Normal	1756	97.56	93.91	93.91	98.48	99.66	99.15	99.15	99.79
202	Afib^f^	2133	98.87	97.19	97.19	99.30	99.51	98.78	98.78	99.70
223	VT^g^	2604	97.45	93.63	93.63	98.41	98.54	96.35	96.35	99.09
12006	Normal	60,867	99.87	99.66	99.66	99.92	99.90	99.76	99.76	99.94
12007	Normal	75,805	99.87	99.66	99.66	99.92	99.99	99.97	99.97	99.99
12008	Normal	78,866	99.94	99.84	99.84	99.96	99.98	99.96	99.96	99.99
12010	SVT^h^ and VT	75,914	99.89	99.72	99.72	99.93	99.97	99.94	99.94	99.98
12011	Bigeminy	66,229	99.92	99.79	99.79	99.95	99.71	99.27	99.27	99.82
12012	Normal	63,820	99.98	99.94	99.94	99.99	99.99	99.99	99.99	99.99
12358	Noise	85,091	99.97	99.92	99.92	99.98	99.96	99.90	99.90	99.97

^a^RNN: recurrent neural network.

^b^ACC: accuracy.

^c^PPV: positive predictive value.

^d^SEN: sensitivity.

^e^SPEC: specificity.

^f^Afib: atrial fibrillation.

^g^VT: ventricular tachycardia.

^h^SVT: supraventricular tachycardia.

### Inference Speed

The inference time of the baseline model on GPUs took 15 minutes and 12 seconds. On the other hand, the lightweight model on CPUs took 3 minutes and 1 second (Table 6). Namely, our lightweight model took only 2 milliseconds to process one beat, and this implies that our model is competitive on a CPU-based wearable hardware [30]. The inference speed according to each parameter can be found in the AWS Re:Invent [31]; controlling the sampling rate and adopting fused RNNs each reduced the inference time. This result also demonstrated that replacing Vanilla RNNs to fused RNNs does not change the processes or parameters constituting a network but only accelerates the processes; consequently, the inference speed was improved without loss of accuracy.

**Table 6:** Comparison of accuracies and latencies.

Model	Latency (min)	Accuracy (%)
RNN^a^ in GPUs^b^	15.12	99.72
RNN in CPUs^c^	120	99.80
Fused RNN in CPUs	3.01	99.80

^a^RNN: recurrent neural network.

^b^GPU: graphics processing unit.

^c^CPU: central processing unit.

## Discussion

### Principal Findings

The results showed that both baseline and lightweight models achieved high prediction performances (ie, accuracies of over 99%). The final model, fused RNNs, showed superior performance in both subclasses: supraventricular and ventricular beat. In addition, the reliability of the lightweight model with fused RNNs was supported with a prediction accuracy of over 99% in each subject as well as overall model performance.

### Limitations

The accuracy of the lightweight model with fused RNN for beat classification was high, but there were still incorrect cases. These false cases were caused with specific rhythms, such as supraventricular tachycardia, bigeminy, and paroxysmal atrial fibrillation.

**Figure 6:**
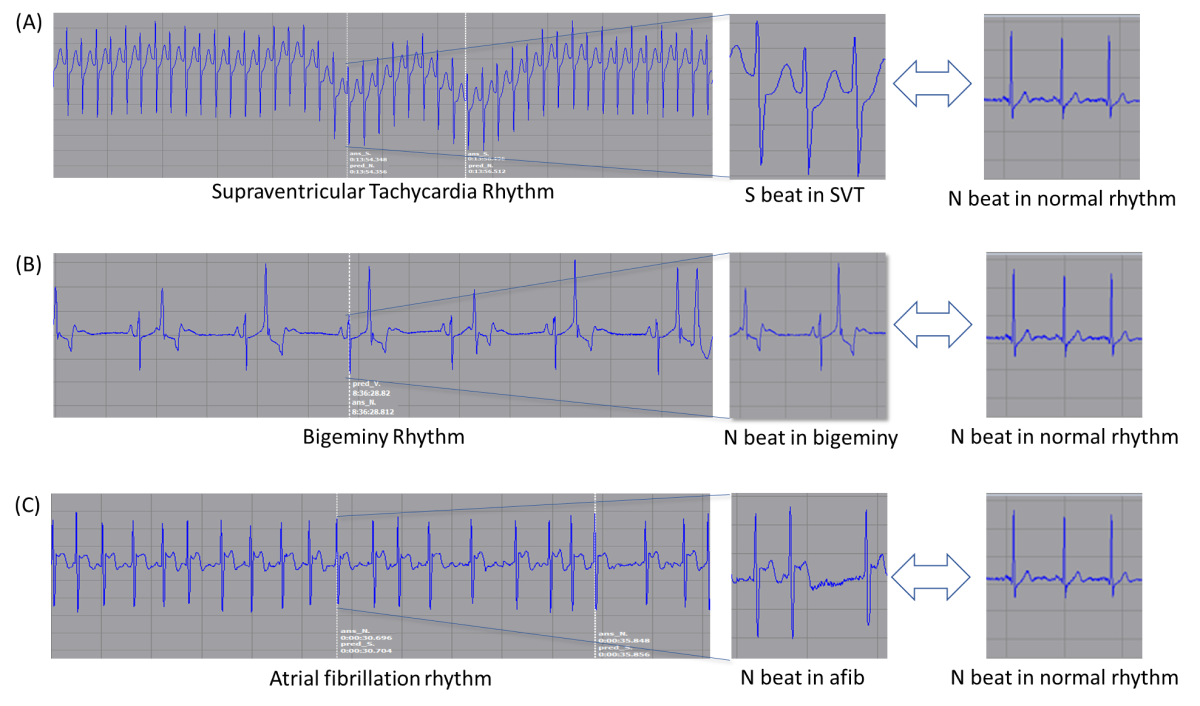
Beats in rhythms with different shapes. SVT: supraventricular tachycardia; S: supraventricular ectopic; N: nonectopic.

We reviewed 1018 beats that were falsely predicted by the lightweight model and interpreted the errors. As a result, the beats composing the abnormal rhythm were often misunderstood (Figure 6). For example, in the case of subject #12010, supraventricular beats in the supraventricular tachycardia rhythm were predicted incorrectly as normal beats. This is because the supraventricular beats in supraventricular tachycardia rhythm have very short intervals between two consecutive beats but have similar morphology to normal beats in normal rhythm (Figure 6A). Supraventricular beats can be easily misjudged as normal beats when the model reviewed only one segment, which has 3 beats. In another example, subject #12011, normal beats in the bigeminy rhythm were predicted as ventricular rhythm. This is because the intervals between normal beats in the bigeminy rhythm are relatively longer due to the leading and trailing ventricular beats. Therefore, normal beats in bigeminy rhythm have a different morphology from those in other rhythms (Figure 6B). The differences in beat morphology were also confirmed in the atrial fibrillation rhythm (Figure 6C).

The case review confirmed that the rhythm affects each ECG beat. Therefore, it is necessary to develop a rhythm model using a wider ECG window. Although Rajpurkar et al [11] recently conducted a classification task for 14 ECG rhythms, most existing studies on rhythm predictions were limited to a specific rhythm such as atrial fibrillation [32,33]. We are developing a universal rhythm prediction model that integrates the results of the beat model developed in this study and other features such as R-R interval and R-peak amplitude.

In our case, the subject-specific evaluation was conducted to demonstrate the reliability of the deep learning models. However, it is necessary to perform an evaluation based on real-world data to support the model’s reliability, which can provide generalized predictions for new data. Currently, this is in progress at a tertiary hospital in South Korea.

### Future Works

The lightweight deep learning model for ECG classification proposed in this paper was adopted as an analysis module for Samsung SDS Cardio. Cardio is a service that collects ECG signals with S-Patch, sends the signals to a cloud-based Web portal for ECG diagnosis, and reports the diagnosis to users through their mobile or gear applications. This service also can be extended to health monitoring for elderly people, who are vulnerable to cardiovascular disease, and for first responders such as firemen. Furthermore, our ECG classifier can be embedded into a health care system together with patient-generated biomedical information analysis (eg, mobile search log, geotagged data) [34] and provide wider and deeper information to users.

### Conclusion

We proposed lightweight deep neural network models that were effective and efficient for ECG beat classification. The proposed models were trained using both the standard Pysionet MIT-BIH database and Samsung S-Patch 2 dataset collected by two major hospitals in New Zealand and South Korea. Our lightweight model with fused RNN achieved a cardiologist-level accuracy of 99.80%. Furthermore, the lightweight model conducted ECG beat predictions on a CPU five times faster than the baseline model with Vanilla RNNs without accuracy loss.

## Abbreviations

AAMI: Association for the Advancement of Medical Instrumentation
ACC: accuracy
BLAS: Basic Linear Algebra Subprogram
CNN: convolutional neural network
CPU: central processing unit
ECG: electrocardiogram (electrocardiographic)
F: fusion beat
FN: false negative
FP: false positive
GEMM: general matrix multiplication
GPU: graphics processing unit
IEC: International Electrotechnical Commission
MIT-BIH: Massachusetts Institute of Technology-Beth Israel Hospital
MKL: Intel Math Kernel Library
N: nonectopic
PPV: positive predictive value
Q: paced or unknown beat
RNN: recurrent neural network
S: supraventricular ectopic
SEN: sensitivity
SPEC: specificity
STFT: short-time Fourier transform
TN: true negative
TP: true positive
V: ventricular ectopic
